# Prospective cohort study on the relationship between long-term air pollution exposure and risk of myocardial infarction

**DOI:** 10.6026/973206300213413

**Published:** 2025-09-30

**Authors:** Rahil Abdusamad Nainam Valappil, Anjaly Mecheril Chandran, Shameela Farha, Sachin Puthusseriyil Shaji, Yash Sharan, Yashita Dharni, Smarin Shaji

**Affiliations:** 1 Department of Critical Care, Iqraa International Hospital, Calicut, Kerala, India; 2 Department of Gastro Medicine, Malankara Orthodox Syrian Church Medical Mission Hospital, Kolenchery, Kerala, India; 3 Department of Emergency Medicine, Calicut Hospital & Nursing home, Calicut, Kerala, India; 4 Department of Emergency Medicine, General Hospital, Muvattupuzha, Kerala, India; 5 Department of Anesthesia and Critical Care, Lok Nayak Hospital, New Delhi, India; 6 Department of General Medicine, Adesh Institute of Medical Sciences and Research (AIMSR), Bathinda, Punjab, India; 7 Department of Traditional Medicine, University of Traditional Medicine, Yerevan, Armenia

**Keywords:** Air pollution, myocardial infarction, cardiovascular risk, PM2.5, prospective cohort studies

## Abstract

The relationship between long-term air pollution exposure and risk of myocardial infarction is of interest. Hence, this five-year prospective cohort study examined 132 adults between the ages of 40 and 65 to determine whether long-term exposure to air pollutants (PM2.5, NO_2_, and SO_2_) was associated with an increased risk of myocardial infarction (MI). Participants who lived in metropolitan regions with high pollution levels had a noticeably higher incidence of MI than those who lived in locations with low exposure levels. According to adjusted risk models, PM2.5 is the most reliable independent predictor of MI incidence. High-exposure individuals also showed significantly higher levels of biomarkers of systemic inflammation. In order to reduce cardiovascular risks, the highlight the necessity of strict air quality restrictions.

## Background:

Heart attacks are the largest cause of death worldwide, and myocardial infarction (MI) is one of the most serious types of cardiovascular diseases (CVDs) [1]. The significant yet underappreciated role that environmental factors particularly air pollution-play in cardiovascular morbidity and death has come to light in recent years [2]. Sulfur dioxide (SO_2_), nitrogen dioxide (NO_2_), and particulate matter (PM2.5) are significant pollutants that are connected to the pathophysiology of endothelial dysfunction, atherosclerosis, systemic inflammation, and plaque rupture-all of which can result in acute coronary events [3]. Urban people are disproportionately exposed to these toxins over an extended period of time due to industrial emissions and excessive traffic [4]. Although several cross-sectional and retrospective studies have implicated a possible link between long-term exposure to air pollution and cardiovascular outcomes, there is currently insufficient prospective data, especially in developing countries [5]. To guide clinical risk evaluations and public health policies, it is imperative to understand this connection [6]. Therefore, this is of interest to bridge this gap by prospectively evaluating the relationship between the incidence of myocardial infarction in a particular adult group and long-term exposure to air pollution.

## Materials and Methods:

This prospective cohort study was conducted over five years in a metropolitan area with different air pollution levels. Following screening for cardiovascular risk factors and exclusion of individuals with a history of myocardial infarction, established coronary artery disease, or chronic pulmonary illnesses, 132 adults aged 40-65 were recruited. Residential exposure levels to PM2.5, NO_2_, and SO_2_ were used to stratify participants. This was done using local environmental monitoring stations and geocoded satellite data. Baseline data included detailed sociodemographic profiles, medical history, lifestyle factors, and occupational exposure. Blood samples were collected to evaluate lipid profiles, high-sensitivity C-reactive protein (hs-CRP), and other inflammatory biomarkers. Participants were followed annually for clinical events, with myocardial infarction confirmed through ECG changes, cardiac enzyme levels, and physician diagnosis. Cox proportional hazards models were used to analyze the association between pollutant exposure levels and incidence of MI, adjusting for age, sex, BMI, smoking status, hypertension, diabetes, and physical activity. Ethical approval was obtained, and informed consent was secured from all participants.

## Results:

Long-term exposure to elevated air pollutants, particularly PM2.5, was significantly associated with increased incidence of myocardial infarction over the 5-year follow-up. Individuals residing in high-exposure zones demonstrated higher inflammatory markers and cardiovascular risk profiles compared to those in low-exposure areas. Table 1 (see PDF) highlights that participants in high-pollution areas had higher BMI and smoking prevalence compared to those in low-pollution zones, suggesting lifestyle-related risk factors were more pronounced in polluted environments. Table 2 (see PDF) confirms that high-exposure zones had significantly elevated PM2.5, NO_2_, and SO_2_ concentrations, validating the environmental stratification and establishing clear exposure differences. Table 3 (see PDF) shows that systemic inflammatory markers, particularly hs-CRP, IL-6, and TNF-alpha, were markedly higher in the high-exposure group, indicating pollution-driven inflammation. Table 4 (see PDF) demonstrates that myocardial infarction incidence was three times higher in the high-exposure group (19.4%) compared to the low-exposure group (6.2%) over the five-year follow-up. Table 5 (see PDF) presents Kaplan-Meier survival analysis, showing significantly worse MI-free survival in the high-exposure group, with divergence becoming more evident over time and statistically significant by year 5. Table 6 (see PDF) indicates that multivariate Cox regression identified PM2.5 as the strongest independent predictor of MI, even after adjusting for other risk factors such as BMI and smoking. Table 7 (see PDF) reveals that the high-exposure group had more atherogenic lipid profiles, with higher LDL and triglycerides but lower HDL levels, reinforcing pollution's metabolic impact. Table 8 (see PDF) shows lower physical activity and greater sedentary time in the high-exposure group, contributing to elevated cardiovascular risk burden. Table 9 (see PDF) reports higher systolic and diastolic blood pressures among high-exposure participants, consistent with vascular stress from pollution. Table 10 (see PDF) demonstrates that subclinical ECG changes such as ST depression and T-wave inversion occurred more frequently in the high-exposure group, suggesting early cardiac effects prior to overt MI.

Table 1 (see PDF) shows Participants in high-pollution areas had higher baseline BMI and smoking rates, which are known cardiovascular risk factors. Table 2 (see PDF) shows High-exposure group had significantly greater levels of key air pollutants, validating the environmental stratification. Table 3 (see PDF) shows Biomarkers of systemic inflammation, particularly hs-CRP and IL-6, were significantly elevated in high-exposure participants. Table 4 (see PDF) shows High exposure group had a greater number of myocardial infarction events during follow-up. Table 5 (see PDF) shows Kaplan-Meier analysis shows significant divergence in MI-free survival between groups over time. Table 6 (see PDF) shows Cox regression analysis revealed PM2.5 as the most significant predictor of MI, even after adjusting for other risk factors. Table 7 (see PDF) shows Lipid profiles were worse in the high-exposure group, indicating a pro-atherogenic state. Table 8 (see PDF) shows Physical activity levels were lower among high-exposure participants, possibly contributing to elevated MI risk. Table 9 (see PDF) shows Blood pressure was marginally higher in the high-exposure group, aligning with pollutant-related vascular stress. Table 10 (see PDF) shows Pollution-exposed individuals had higher rates of subclinical ECG abnormalities during routine follow-up.

## Discussion:

This prospective cohort study provides compelling evidence linking long-term exposure to air pollutants-especially PM2.5-with an elevated risk of myocardial infarction (MI). Over the five-year follow-up, individuals residing in high-exposure zones experienced significantly higher MI incidence, supporting the hypothesis that chronic environmental stress contributes to cardiovascular pathology [7]. Elevated inflammatory markers such as hs-CRP and IL-6 in these individuals further suggest that systemic inflammation may serve as a mechanistic bridge between air pollution and atherothrombosis. These biological alterations were compounded by worse lipid profiles, higher blood pressure, reduced physical activity, and more frequent subclinical ECG changes, all of which amplify cardiovascular risk [8, 9]. While socioeconomic and lifestyle factors partially influenced outcomes, the association between PM2.5 and MI remained significant even after adjusting for confounders. This underscores air pollution not merely as a background risk but as a direct, modifiable threat to cardiovascular health [10]. The findings align with and extend prior literature by demonstrating that environmental exposures influence clinical endpoints over time-not just surrogate markers. Importantly, this study highlights the hidden burden of pollution-related cardiovascular disease in urban populations, especially in developing nations where air quality regulations are often poorly enforced [11]. Integrated evidence from cohort studies supports the hypothesis that long-term exposure to PM2.5 is a risk factor for MI [12]. Future interventions should prioritize both pollution control and individual risk reduction strategies to mitigate this growing public health concern.

## Conclusion:

Long-term exposure to elevated air pollution, particularly PM2.5, significantly increases the risk of myocardial infarction through pathways involving systemic inflammation and metabolic dysregulation. Urban populations are especially vulnerable due to sustained pollutant exposure and associated lifestyle factors. Targeted environmental and clinical interventions are essential to reduce pollution-related cardiovascular morbidity.
